# Anthelmintic effects of some medicinal plants on different life stages of *Fasciola hepatica*: Evidence on oxidative stress biomarkers, and DNA damage

**DOI:** 10.1371/journal.pntd.0012251

**Published:** 2024-06-17

**Authors:** Mohaddeseh Allahyari, Farnaz Malekifard, Mohammad Yakhchali

**Affiliations:** Department of Pathobiology, Faculty of Veterinary Medicine, Urmia University, Urmia, Iran; KARI-Trypanosomiasis Res Centre, KENYA

## Abstract

Fasciolosis caused by *Fasciola hepatica* is a major public health and economic problem worldwide. Due to the lack of a successful vaccine and emerging resistance to the drug triclabendazole, alternative phytotherapeutic approaches are being investigated. This study investigated the *in vitro* anthelmintic activity of Lavender (*Lavandula angustifolia*) and carob (*Ceratonia siliqua L*.) essential oils (EOs) against *F*. *hepatica*. The *in vitro* study was based on an egg hatch assay (EHA), adult motility inhibition assays, DNA damage, reactive oxygen species (ROS) level along with several oxidative stress biomarkers including glutathione peroxidase (GSH), and glutathione-S-transferase (GST), superoxide dismutase (SOD) and malondialdehyde (MDA). To this end, different concentrations of *L*. *angustifolia* and *C*. *siliqua* EOs (1, 5, 10, 25 and 50 mg/mL) were used to assess anthelmintic effects on different life stages including egg, and adults of *F*. *hepatica* for 24 hrs. The results indicated that these EOs play a significant role as anthelminthics, and the effect was dependent on time and concentration. The *in vitro* treatment of *F*. *hepatica* worms with both *L*. *angustifolia* and *C*. *siliqua* EOs increased DNA damage, ROS production and induction of oxidative stress (decreased SOD, GST and GSH, and increased MDA), significantly compared to control. Therefore, it can be concluded that *L*. *angustifolia* and *C*. *siliqua* EOs have the potential to be used as novel agents for the control and treatment of *F*. *hepatica* infections. Further studies are required to investigate their pharmacological potential and effectiveness *in vivo* for the treatment of parasitic infections.

## Author summary

Fasciolosis is a disease caused by a leaf-shaped trematode called *Fasciola hepatica*. This disease is becoming increasingly common in humans and has been reported on five continents. *F*. *hepatica* primarily infects the bile ducts of cattle, sheep and goats, causing significant economic losses to the global livestock industry. Unfortunately, this disease has been neglected and there is currently no commercially viable vaccine to prevent it. The main treatment for this disease is chemotherapy, with triclabendazole (TCBZ) being the drug of choice for controlling these parasites. However, there is growing concern about the increase in reports of drug resistance to TCBZ in parasites. Therefore, there is an urgent need to find alternative treatment methods. Natural herbal products give great hope as they contain a large reservoir of medicinal ingredients that are effectively used against various parasitic diseases. The aim of the current study is to evaluate the *in vitro* effect of essential oils of lavender (*Lavandula angustifolia*) and carob (*Ceratonia siliqua L*.) on *F*. *hepatica*. According to the findings of this study, *L*. *angustifolia* and *C*. *siliqua* essential oils (EOs) have significant effects as anthelmintics. The use of *L*. *angustifolia* and *C*. *siliqua* EOs *in vitro* significantly increased DNA damage, ROS production, and oxidative stress in *F*. *hepatica* worms. Therefore, it can be concluded that these EOs have the potential to be used as novel agents for the control and treatment of *F*. *hepatica*.

## Introduction

Fasciolosis is a disease caused by a leaf-shaped trematode called *Fasciola hepatica*. This disease is becoming increasingly common in humans, with cases now reported on five continents [1]. The World Health Organization (WHO) estimates that over 2.4 million people in more than 70 countries are infected with the disease. *F*. *hepatica* mainly affects the bile ducts of cattle, sheep, and goats, causing significant economic losses [2].

The main treatment for this disease is chemotherapy as there is currently no vaccine available [3]. The drug of choice for controlling these flukes is triclabendazole (TCBZ), which eliminates both juvenile and adult flukes by disrupting their β-tubulin polymerization (Fairweather and Boray, 1999). However, there is an increase in reports of drug resistance to TCBZ in flukes, which is a cause for concern. Therefore, there is a growing need to find alternative treatment methods [4].

There are several factors that can influence the epidemiological pattern of fascioliasis, such as drug resistance to fascioliasis, human-induced changes in the environment and climate change [5]. It is important for the health of humans and animals to get this disease under control. However, the effectiveness of current disease control measures is decreasing in many infected areas. Alternative strategies to control fasciola include pasture management, biological control, and the use of antifasciolid drugs to treat fascioliasis or its effects [6].

Research suggests that chemotherapy procedures are inefficient in controlling infections and often result in the selection of resistant lineages of *Fasciola* spp. worldwide [7]. Due to high veterinary costs, limited availability of antiparasitic chemical compounds, drug resistance, as well as the presence of drugs in milk and associated toxicity, the study of traditional properties of herbs as an alternative treatment is warranted [8–10].

Numerous traditional medicines and novel drugs have been derived from plants and tested for their antiparasitic properties both *in vitro* and *in vivo* [11]. Several studies have been conducted with lavender (*Lavandula angustifolia*) and carob (*Ceratonia siliqua L*.). These studies showed that these plants possess antipsoriatic, antitoxoplasmotic, antidiabetic and antidiarrheal properties [12–15]. They have also been shown to exhibit antiparasitic activity under both *in vivo* and *in vitro* conditions [16–20]. Both *Lavandula angustifolia* and *Ceratonia siliqua* affect multiple signaling molecules and different metabolic pathways [20,21].

To our knowledge, there is no information on the potential anthelminthic effects of *L*. *angustifolia* and *C*. *siliqua* essential oils (EOs) on trematodes, particularly flukes. Therefore, this study designed to assess the anthelminthic effects of *L*. *angustifolia* and *C*. *siliqua* EOs by measuring various parameters such as egg hatching and adult worm motility. In addition, we investigated the effect of the *L*. *angustifolia* and *C*. *siliqua* EOs on the development of oxidative stress by measuring several biomarkers of oxidative markers, including reactive oxygen species (ROS), superoxide dismutase (SOD), glutathione peroxidase (GSH), glutathione-S-transferase (GST), and malondialdehyde (MDA) and DNA damage, using *in vitro* approaches.

## Methods

### Ethical compliance

Ethical considerations for the study was approved by Animal Ethics Committee in Urmia University, Urmia, Iran (IR-UU-AEC-3/63) and conducted under the regulations of this committee.

### Essential Oils

The plants *L*. *angustifolia* and *C*. *siliqua* were purchased from a Persian herbal market and confirmed by the Natural Resource Center. The plants were dried in the shade at a temperature of 25–30°C for one week. They were then chopped using an electric mixer. The essential oil was extracted from the plants using hydrodistillation. One hundred grams of each plant was ground and placed in a distillation flask with 900 mL of water. The flasks were heated at 100°C for 3 hrs using a Clevenger apparatus. The essential oil extract was isolated from the top of the Clevenger device. This process was repeated several times to obtain enough essential oils. Anhydrous sodium sulfate was used to dry the essential oils obtained. The dried oils were filtered and placed in amber bottles for storage until analysis. The bottles were stored at 4°C. The yield of essential oils (EOs) was measured by weighing the obtained essential oils each time and reported as a percentage of EOs per 100 g of plants [19,20].

### Analysis of the Composition of Essential Oils

Gas chromatography-mass spectrometry was used to analyze the chemical composition of the EOs using a Thermo Scientific instrument. Helium was used as the carrier gas and the splitting ratio was set to 0.50 mL per minute. Gas spectrometry conditions included increasing the oven temperature from 40 to 250°C in 3 min at a rate of 80°C per min. The temperature of the detector and injector was set to 250°C. The compounds present in essential oils were identified by comparing their relative retention time with a measurement database on a capillary column and matching their peak mass spectra with those from authentic samples and published data [20,22].

### Parasite collection

The adult flukes of *F*. *hepatica* were collected from the bile duct and gallbladder of cattle slaughtered at the local slaughterhouse in Urmia city, Iran. They were rinsed thoroughly in Hanks’ balanced salt solution before being incubated separately in RPMI 1640 medium (Sigma-Aldrich Chemie GmbH, Germany) containing different concentrations of *L*. *angustifolia* and *C*. *siliqua* EOs [3]. Only intact and actively motile worms were used immediately for this study.

### Collection and extraction of *F*. *hepatica* eggs

The technique used by Moazeni and Khademolhoseini (2016) [23] was used to extract *F*. *hepatica* eggs from the gallbladders of cattle naturally infected with *F*. *hepatica*. The bile was transferred to glass cylinders aseptically and allowed to harden for 30 min. The eggs settled at the bottom of the cylinders and the remaining liquid was removed. The eggs were then washed several times with normal saline. Finally, they were stored in a dark glass container with normal saline at 4°C for later use.

### EOs suspension preparation

Four different concentrations of EOs (1, 5, 10, 25 and 50 mg/mL) were prepared in RPMI 1640 medium supplemented with 5% (v/v) fetal bovine serum and 10 mL/L Penicillin—Streptomycin solution.

### Egg Hatch Test

In this study, *F*. *hepatica* eggs were exposed to different concentrations of *L*. *angustifolia* and *C*. *siliqua* EOs (1, 5, 10, 25 and 50 mg/mL) at various times (24, 48 and 72 hrs). For each experiment, a drop of egg-rich sediment containing at least 1,500 eggs was added to a test tube containing 10 mL of each EOs. The exact number of eggs was counted using an optical microscope. The tubes were then incubated at 37°C for 24, 48 and 72 hrs. Afterwards, 9 mL of the upper part of the solution was removed, avoiding the settled eggs. The eggs were then washed and transferred to small plastic containers containing 5 mL of dechlorinated tap water. The containers were incubated at 28°C for 14 days, and at the same time a control group of at least 3,000 eggs without exposure to EOs was also incubated at 28°C. At the end of the incubation period, eggs were streaked on a manually scaled glass slide, covered with a coverslip, and examined under a light microscope. The ovicidal activity of the EOs was determined by counting a minimum of 1,000 eggs in each experiment. The experiment was repeated three times for each concentration [23].

### *In vitro* treatment of parasites

To examine the *in vitro* effect of EOs on adult *F*. *hepatica* worms, a total of 10 worms were cultured in triplicate in 5 mL of RPMI medium supplemented with 5% (v/v) fetal bovine serum containing different concentrations of EOs were incubated for 24 hrs at 37 ± 1°C. Triclabendazole (TCBZ 20 μg/mL) and PBS were included in the test as positive and negative controls, respectively. The adult *F*. *hepatica* was exposed for 24 hrs and then rinsed with phosphate-buffered saline. Parasites were homogenized in 100 mM Tris-HCl buffer, pH 7.4, centrifuged at 10,000 × *g* for 30 min at 4°C, and the supernatant was collected and stored at − 80°C until use [3].

### Observation on parasite mortality and mobility

After incubating worms in different EOs concentrations, the parasites mortality and mobility was monitored every 4 hrs for up to 24 hrs under experimental conditions. The mobility of control worms (without EOs) was also recorded. Using a dissecting microscope (SMZ1270, Nikon, Tokyo, Japan), at 2x magnification, the number of motile (live) and immotile (dead) worms was counted and recorded separately for each concentration. A 5-grade qualitative scale was used to assess parasite mobility. The experiment was repeated three times before the results were presented as a percentage of mortality. Percent mortality was calculated for each concentration using following formula [24]:

Mortality (%) = (number of dead worms/total number of worms per test) × 100

### Reactive oxygen species estimation

The amount of superoxide anions produced when treating worms with *L*. *angustifolia* and *C*. *siliqua* EOs was determined according to the method of Sim Choi et al. (2006) [25]. Briefly, the treated and untreated samples were incubated in a 2% nitroblue tetrazolium (NBT) solution at 37 ± 1°C for 2 hrs. The formed formazan crystals were then dissolved in DMSO before absorbance was recorded at 620 nm.

### Glutathione Peroxidase assay

The GSH detection kit (Ransel, RanDox Co., UK) was used to determine GSH activity. The measurement method described by the manufacturer was followed and the absorption reduction was measured spectrophotometrically using a blank sample at 340 nm [26]. The protein content of the supernatant was measured using the Lowry colorimetric method and bovine serum albumin (BSA) was used as a standard. It should be noted that the units are classified based on the protein content of the parasite homogenate [27]

### Glutathione-S-transferase assay

The GST assay was performed according to the method described by Habig et al. (1974) [28]. The assay used 10 mM GSH and 1 mmol CDNB (1-chloro-2,4-dinitrobenzene) as substrate. To start the assay, 50 μL of protein sample was added to 100 mM potassium phosphate buffer (pH 6.5). Enzyme activity was calculated as nmol of CDNB conjugate formed per minute per milligram of protein. A molar extinction coefficient of 9.6x103 M/cm was used to calculate the enzyme activity.

### Estimation of superoxide dismutase (SOD) activity

To determine SOD activity, we used a standard commercial kit (RanSod, RanDox Co., UK) and performed the xanthine-xanthine oxidase assay [29]. The SOD activity was recorded at the wavelength of 505 nm using a standard curve.

### Assessment of lipid peroxidation (MDA)

To measure MDA as a biomarker of lipid peroxidation, a method from that described by Buege and Aust was used [30]. For this purpose, one volume of homogenate was thoroughly mixed with two volumes of a stock solution consisting of 15% v/v trichloroacetic acid, 0.375% v/v thiobarbituric acid and 0.25 mol/L hydrochloric acid. After the heating and cooling periods, the resulting solution was centrifuged at 1000 rpm for 10 min to obtain a clear solution. The absorbance at 535 nm was determined and the MDA content was calculated using 1.56 × 105 mol/cm as the molar absorption coefficient. MDA content was recorded in nmol per mg protein.

### DNA damage assessment

A modified version of the alkaline comet assay [31] was used to assess DNA damage in *F*. *hepatica*. The non-invasive extrusion method was employed to collect the coelomocytes of the worms after incubation [32].The comets were visually inspected and scored based on the amount of DNA in their tails [33].The images were grouped based on the fluorescence intensity in the comet tail and assigned a score of 0, 1, 2, 3, or 4. Total scores were expressed in arbitrary units ranging from 0 to 400 [34].

### Statistical analysis

Statistical analysis was performed using SPSS software (version 26, Chicago, IL, USA). The homogeneity of variances was tested using the Levene test. To compare the analyzed parameters between control and treatment groups, one-way and two-way ANOVA as well as the Bonferroni post hoc test were used. Data were presented as mean ± SD (standard deviation) and a *p* value less than 0.05 (*p* ≤ 0.05) was considered statistically significant.

## Results

### Chemical Components of Essential Oils

According to gas chromatography-mass spectrometry (GC/MS) analysis of essential oils, lavender oil contained borneol (29.7%), linalool (26.20%) and alpha-pinene (14.30%) as main chemical components (as shown in Table 1). On the other hand, *C*. *siliqua* oil contains nonadecane (23.34%), 1,2-benzenedicarboxylic acid, dibutyl ester (15.95%), heneicosan (14.61%), eicosene (9.66%), 1,2-benzenedicarboxylic acid (7.51%) and b-cedrene (7.37%) as the main chemical components (as shown in Table 1).

**Table 1:** Major chemical compounds in *L*. *angustifolia and C*. *siliqua EOs* identified by GC-MS.

Essential Oils	Major compounds	Percent
***Lavandula angustifolia***	Ocimene	7.82
	Camphor	4.55
	Linalyl acetate	5.60
	Borneol	22.70
	1,8-cineol	11.50
	ɑ-pinene	14.30
	linalool	26.20
***Ceratonia siliqua***	Nonadecane	23.34
	Heneicosane	14.61
	β-Cedrene	7.37
	Camphor	5.18
	1,2-Benzenedicarboxylic acid, dibutyl ester	15.95
	Eicosene	9.66
	1,2-Benzenedicarboxylic acid	7.51
	β-Patchoulene	3.47
	Other	12.91

### Adult worm mobility test

Exposure to different concentrations of EOs from *L*. *angustifolia* and *C*. *siliqua* (1, 5, 10, 25, and 50 mg/mL) for 24 hrs resulted in significant inhibition of motility in adult worms, and the inhibition rate was higher in adult worms compared to negative controls (Table 2). It should be noted that the inhibition rate depends on the exposure time and EOs dose. In the present study, 50 mg/mL *L*. *angustifolia* EOs completely inhibited the mobility of adult worms during the first 12 hrs of observation. The same effects were observed for 50 mg/mL *L*. *angustifolia* and *C*. *siliqua* EOs during the 16 hrs observation period (Table 2).

**Table 2:** The effect of various concentrations and incubation time of *Lavandula angustifolia* and *Ceratonia siliqua* EOs on the motility of *Fasciola hepatica*.

Hours	Control (-)	Triclabendazole (control +)	*Lavandula angustifolia*					*Ceratonia siliqua*				
			1 mg/mL	5 mg/mL	10 mg/mL	25 mg/mL	50 mg/mL	1 mg/mL	5 mg/mL	10 mg/mL	25 mg/mL	50 mg/mL
**0 hrs**	++++	++++	++++	++++	++++	++++	++++	++++	++++	++++	++++	++++
**4 hrs**	++++	++++	++++	++++	++++	++++	+++	++++	++++	++++	++++	+++
**8 hrs**	++++	++++	++++	++++	+++	+++	++	++++	++++	+++	+++	+++
**12 hrs**	++++	+++	++++	+++	+++	++	-	++++	++++	+++	++	+
**16 hrs**	++++	++	++++	+++	++	+	-	++++	+++	++	+	-
**20 hrs**	++++	-	+++	++	-	-	-	+++	++	+	-	-
**24 hrs**	+++	-	++	-	-	-	-	++	-	-	-	-

++++ (high), +++ (moderate), ++ (low), + (very low),—(no motility)

### Adult worm mortality test

According to the results presented in Table 3, it was observed that increasing the concentration of *L*. *angustifolia* and *C*. *siliqua* EOs and the exposure time resulted in the destruction of adult worms. The adult worms exposed to lower concentrations (1 and 4 ppm) showed no adverse effects in the first 4 hrs interval. However, the other higher concentrations were able to destroy the adult worms within 4 hrs. In this study, it was observed that the highest concentration (50 mg/mL) of *L*. *angustifolia* EOs caused 100% mortality within the first 12 hrs of observation. According to Table 3, 100% mortality was observed in the positive controls within 20 hrs of the start of observation. In contrast, the mortality rate for negative control was approximately 7.87% after 24 hrs.

**Table 3:** The effect of various concentrations and incubation time of *Lavandula angustifolia* and *Ceratonia siliqua* EOs on the mortality of *Fasciola hepatica*.

Hours	Control (-)	Triclabendazole (control +)	*Lavandula angustifolia*					*Ceratonia siliqua*					*P* value
			1 mg/mL	5 mg/mL	10 mg/mL	25 mg/mL	50 mg/mL	1 mg/mL	5 mg/mL	10 mg/mL	25 mg/mL	50 mg/mL	
**0 hrs**	0.0±0.0 ^Ab^	0.0±0.0 ^Ae^	0.0±0.0 ^Ad^	0.0±0.0 ^Af^	0.0±0.0 ^Ae^	0.0±0.0 ^Ae^	0.0±0.0 ^Ad^	0.0±0.0 ^Ad^	0.0±0.0 ^Ae^	0.0±0.0 ^Af^	0.0±0.0 ^Af^	0.0±0.0 ^Ae^	-
**4 hrs**	0.0±0.0 ^Fb^	7.56±1.21 ^Ed^	0.0±0.0 ^Fd^	0.87±0.51 ^Ff^	12.73±1.19 ^Dd^	27.05±1.67 ^Bd^	40.09±1.61 ^Ac^	0.0±0.0 ^Fd^	0.0±0.0 ^Fe^	11.65±0.47 ^De^	19.95±1.13 ^Ce^	28.65±1.20 ^Bd^	*p*<0.001
**8 hrs**	0.0±0.0 ^Gb^	10.42±0.13 ^Ed^	0.0±0.0 ^Gd^	6.04±2.33 ^Fe^	15.45±1.47 ^Dd^	54.65±0.55 ^Bc^	67.44±0.94 ^Ab^	0.0±0.0 ^Gd^	2.03±0.37 ^Ge^	17.45±1.63 ^Dd^	39.54±1.07 ^Cd^	59.65±1.39 ^Bc^	*p*<0.001
**12 hrs**	0.0±0.0 ^Ib^	31.12±0.45 ^Ec^	8.56±0.35 ^Hc^	20.07±1.50 ^Fd^	34.56±0.83 ^Ec^	70.65±2.78 ^Cb^	100.0±0.0 ^Aa^	7.56±2.61 ^Hc^	15.34±1.29 ^Gd^	32.05±2.37 ^Ec^	63.45±1.42 ^Dc^	90.54±2.60 ^Bb^	*p*<0.001
**16 hrs**	5.55±0.45 ^Fa^	67.45±0.61 ^Cb^	10.08±0.28^Ec^	41.44±1.67 ^Dc^	69.12±2.45 ^Cb^	98.65 ±0.39^Aa^	100.00±0.0 ^Aa^	11.45±2.57^Eb^	39.05±1.83 ^Dc^	67.65±1.47 ^Cb^	90.54±2.12 ^Bb^	100.00±0.0 ^Aa^	*p*<0.001
**20 hrs**	6.03±0.40 ^Da^	100.00±0.0 ^Aa^	15.67±0.40^Cb^	74.34±1.83 ^Bb^	100.00±0.0 ^Aa^	100.00±0.0 ^Aa^	100.00±0.0 ^Aa^	14.99±1.49^Cb^	72.56±1.55 ^Bb^	98.9±2.10 ^Aa^	100.00±0.0 ^Aa^	100.00±0.0 ^Aa^	*p*<0.001
**24 hrs**	8.46±0.46 ^Ca^	100.00±0.0 ^Aa^	20.4±2.74 ^Ba^	100.00±0.0 ^Aa^	100.00±0.0 ^Aa^	100.00±0.0 ^Aa^	100.00±0.0 ^Aa^	19.04±0.48 ^Ba^	100.00±0.0 ^Aa^	100.00±0.0 ^Aa^	100.00±0.0 ^Aa^	100.00±0.0 ^Aa^	*p*<0.001
***P* value**	*p*<0.001	*p*<0.001	*p*<0.001	*p*<0.001	*p*<0.001	*p*<0.001	*p*<0.001	*p*<0.001	*p*<0.001	*p*<0.001	*p*<0.001	*p*<0.001	

Different superscripts (a-f) within the same column indicate a significant toxicity effect of each concentration of EOs within different exposure time. Different superscripts (A-I) within the same row indicate a significant toxicity effect of different concentration of EOs during each exposure times.

### Egg Hatch Test

Table 4 shows that *L*. *angustifolia* and *C*. *siliqua* EOs have significant ovicidal activity. At 25 and 50 mg/mL in 24 hrs, *L*. *angustifolia* had a higher percentage of inhibition (100%) (Table 4). Similar effects were observed for the 50 mg/mL *C*. *siliqua* essential oils during the 24 hrs observation (Table 4). Study results after 48 hrs showed that 10, 25 and 50 mg/mL of *L*. *angustifolia* and 25 and 50 mg/mL of *C*. *siliqua* were effective in preventing egg hatching by 100% (Table 4). In addition, the results showed that 5, 10, 25 and 50 mg/mL of *L*. *angustifolia* and 10, 25 and 50 mg/mL of *C*. *siliqua* were able to inhibit egg hatching 72 hrs after the experiment (Table 4).

**Table 4:** Inhibitory effect of *Lavandula angustifolia* and *Quercus infectoria* EOs on egg hatch tests against *Fasciola hepatica*.

Test	Hours	Control (-)	*Lavandula angustifolia*					*Ceratonia siliqua*					*P* value
			1 mg/mL	5 mg/mL	10 mg/mL	25 mg/mL	50 mg/mL	1 mg/mL	5 mg/mL	10 mg/mL	25 mg/mL	50 mg/mL	
**Inhibition of egg hatch (%)**	**24 hrs**	2.19±0.08 ^Gc^	44.25±1.05 ^Ec^	68.92±2.43 ^Cc^	98.72±2.27^Aa^	100.00±0.00^Aa^	100.00±0.00^Aa^	36.28±1.41 ^Fc^	54.76±1.51 ^Dc^	89.63±2.33 ^Bb^	99.54±2.28 ^Aa^	100.00±0.00^Aa^	*p*<0.001
	**48 hrs**	11.34±0.26^Eb^	52.63±1.23 ^Db^	89.95±2.31 ^Bb^	100.00±0.00^Aa^	100.00±0.00^Aa^	100.00±0.00^Aa^	49.43±1.57 ^Db^	81.40±2.18 ^Cb^	98.21±2.96 ^Aa^	100.00±0.00^Aa^	100.00±0.00^Aa^	*p*<0.001
	**72 hrs**	21.5±0.34 ^Da^	68.09±1.59 ^Ba^	100.00±0.00^Aa^	100.00±0.00^Aa^	100.00±0.00^Aa^	100.00±0.00^Aa^	63.78±1.22 ^Ca^	98.65±2.46 ^Aa^	100.00±0.00 ^Aa^	100.00±0.00^Aa^	100.00±0.00^Aa^	*p*<0.001

- Different superscripts (a-c) within the same row column indicate a significant inhibitory effect of each concentration of EOs within different exposure time. Different superscripts (A-D) within the same row indicate a significant inhibitory effect of different concentration of EOs during each exposure times.

### Generation of ROS

To measure ROS generation in the worms, the amount of superoxide anions produced upon treatment with *L*. *angustifolia* and *C*. *siliqua* EOs was measured. A concentration-dependent increase in cellular ROS production was observed in worms treated with *L*. *angustifolia* and *C*. *siliqua* EOs (Table 5). This was evidenced by increased absorption levels compared to control worms.

**Table 5:** The effect of various concentrations of *Lavandula angustifolia* and *Ceratonia siliqua* EOs on oxidative stress parameters and DNA damage after 24 hrs.

Test	Control (-)	*Lavandula angustifolia*					*Ceratonia siliqua*					*P* value
		1 mg/mL	5 mg/mL	10 mg/mL	25 mg/mL	50 mg/mL	1 mg/mL	5 mg/mL	10 mg/mL	25 mg/mL	50 mg/mL	
**SOD (U/mg Pro)**	1.67±0.52 ^EF^	1.87±0.56 ^D^	2.43±1.54 ^A^	2.21±0.02 ^B^	1.61±0.32 ^F^	1.23±1.32 ^H^	1.75±1.45 ^E^	2.21±0.54 ^B^	2.01±1.34 ^C^	1.98±0.09 ^C^	1.34±1.81^G^	*p*<0.001
**ROS (absorbance@620 nm)**	1.02±0.67 ^F^	1.08±0.54 ^F^	1.21±1.44 ^E^	1.23±2.43 ^DE^	1.65±1.16 ^B^	1.89±2.42 ^A^	1.09±1.08 ^F^	1.19±0.15 ^E^	1.29±1.34 ^D^	1.52±0.63 ^C^	1.73±1.54 ^B^	*p*<0.001
**GSH (U/mg Pro)**	28.45±0.24 ^A^	27.55±0.25 ^A^	25.32±0.56 ^B^	22.65±0.22^CD^	18.23±0.32^EF^	16.21±0.43 ^F^	27.43±0.23 ^A^	26.25±1.14^AB^	23.43±0.24 ^C^	20.6±1.18 ^DE^	18.76±0.12^EF^	*p*<0.001
**MDA (nmol/mg Pro)**	0.34±0.07 ^E^	0.35±0.56 ^E^	0.47±0. 34 ^D^	0.73±1.35 ^C^	0.84±0.34 ^B^	0.99±1.43 ^A^	0.35±0.45 ^E^	0.49±1.54 ^D^	0.79±0.32 ^BC^	0.84±1.44 ^B^	0.95±1.15 ^A^	*p*<0.001
**GST (U/mg Pro)**	33.09±0.16 ^A^	32.4±0.15 ^AB^	31.4±0.67 ^BC^	30.8±2.66 ^CD^	28.9±0.05 ^D^	24.4±1.23 ^E^	32.85±1.43^AB^	31.5±0.54 ^BC^	30.4±1.65 ^CD^	29.76±1.43^CD^	25.8±0.65 ^E^	*p*<0.001
**DNA damage (nmol/mg Pro)**	4.34±0.55 ^D^	5.54±1.24 ^CD^	7.54±1.67 ^C^	15.5±1.56 ^B^	19.45±1.66 ^A^	21.87±1.32 ^A^	4.56±1.56 ^D^	7.76±1.15 ^C^	13.05±2.65 ^B^	18.65±1.25 ^A^	20.76±2.08 ^A^	*p*<0.001

-SOD: Superoxide dismutase; GSH: Glutathione peroxidase; MDA: Malondialdehyde; ROS: Reactive Oxygen Species; GST: Glutathione-S-transferase. Different superscripts (A-H) within the same row indicate a significant effect.

### Superoxide dismutase activity

It was found that the activity of SOD, the main antioxidant enzyme of *F*. *hepatica* worms, which is responsible for modulating oxidative stress by metabolizing the ROS generated in the flukes, was significantly reduced (*p* ≤ 0. 05). The higher concentrations of 50 mg/mL of the EOs from *L*. *angustifolia* and *C*. *siliqua* produced a maximum inhibitory effect, while the lowest concentration (5, 10 mg/mL) caused an increase in SOD activity (Table 5).

### Measurement of GSH activity

The content of GSH was significantly reduced upon treatment with *L*. *angustifolia* and *C*. *siliqua* EOs. As shown in Table 5, the activity of GSH decreased significantly after exposure to different concentrations of *L*. *angustifolia* and *C*. *siliqua* EOs (*p* ≤ 0. 05).

### Glutathione-S-transferase activity

A significant decrease in the specific activity of GST was observed when the worms were treated with higher concentrations of 20 and 50 mg/mL *L*. *angustifolia* and *C*. *siliqua* EOs (Table 5).

### Assessment of lipid peroxidation

The content of MDA, a major end product of the lipid peroxidation process that occurs under oxidative stress, was found to increase in a concentration-dependent manner. Although there was no significant change in MDA level at the lowest concentration (1 mg/mL), a significant increase (*p* ≤ 0. 05) in MDA level was associated with an increase in the concentration of *L*. *angustifolia* and *C*. *siliqua* EOs observed compared to control worms (Table 5).

### DNA damage

The DNA damage of *F*. *hepatica* was assessed in the tail DNA. As shown in Table 5, the concentration of EOs from *L*. *angustifolia* and *C*. *siliqua* affected DNA damage compared to negative controls. The highest concentration of EOs (50 mg/mL) increased damage by approximately fivefold compared to negative controls.

## Discussion

Fasciolosis is a disease that affects cattle, sheep and goats and has a significant economic impact on the global livestock industry [35]. Unfortunately, this disease has been neglected and there is still no effective and commercially viable vaccine to prevent it. While the flukicide triclabendazole (TCBZ) has been used successfully against *Fasciola* species, the emergence of drug resistance combined with the high zoonotic potential of flukes has made control of fasciolosis more difficult [4]. Due to the increasing ineffectiveness of TCBZ, researchers are forced to look for alternatives to combat parasites. Natural herbal products offer great hope because they contain a large reservoir of ingredients with medicinal properties [36]. These natural products have been used effectively against a variety of parasitic diseases by boosting viability and egg production, reducing worm burden, altering antioxidant enzyme levels, and additionally inducing worm apoptosis [37–39]. Regarding the oxidative stress induction by *L*. *angustifolia* and *C*. *siliqua* EOs in living organisms [20,21], The current study aimed at evaluating the effect of EOs from *L*. *angustifolia* and *C*. *siliqua* on the development of oxidative stress in adult *F*. *hepatica* after exposure to different concentrations of EOs. In the present study, egg hatchability and motility of adult worms were also semi quantitatively assessed.

This study examined the potential inhibitory effect of EOs from *L*. *angustifolia* and *C*. *siliqua* on eggs of *F*. *hepatica*. According to the current study, exposure to 5, 10, 25, and 50 mg/mL EOs of *L*. *angustifolia* and 10, 25, and 50 mg/mL EOs of *C*. *siliqua* for 72 hrs inhibited the hatching of *F*. *hepatica* eggs. Various herbal plants have been reported to have ovicidal activity against *F*. *hepatica* eggs. For example, Moazeni and Khademolhoseini (2016) [23] demonstrated the ovicidal effect of the methanolic extract of *Zingiber officinale* on eggs of *F*. *hepatica* in an *in vitro* study. Their study found that 100% ovicidal efficacy was achieved with ginger extract at concentrations of 5 and 10 mg/mL for treatment durations of 48 and 24 hrs, respectively. Marques et al. (2020) [40] also reported that *Eugenia uniflora*, *Harpagohytum procumbens*, *Psidium guajava L*. and *Stryphnodendron astringens* showed high efficacy in controlling egg hatching in *F*. *hepatica* eggs at the doses used. In another *in vitro* study, Pereira et al. (2016) [41] reported the fasciolicidal effect of *Momordica charantia* extract on the eggs of *F*. *hepatica* liver fluke at different concentrations and at different times.

For a long time, worm motility was considered an important factor in testing the anthelmintic effects of various medications and natural products. This is because the worms need to search for suitable microhabitats and feed within the host, which is a key aspect of worm physiology [42]. In the current study, *F*. *hepatica* motility was found to be reduced in a concentration and time-dependent manner. A 16 h exposure to the highest concentration (50 mg/mL) of both EOs completely destroyed adult *F*. *hepatica*. Therefore, the marked decrease in worm motility after treatment with EOs from *L*. *angustifolia* and *C*. *siliqua* likely to significantly reduce the invasive potential of the migratory flukes, as suggested by Rehman (2017) for another digenetic trematode, *Clinostomum complanatum* [43]. Similar results were reported in a study by Alvarez-Mercado et al. in which five plant extracts, including *Lantana camara*, *Bocconia frutescens*, *Piper auritum*, *Artemisia mexicana and Cajanus cajan*, showed promising fascioliscid activity *in vitro*[44].

Oxidative stress is harmful to worms because it can alter the macromolecules in their cells, which can alter the normal function of important enzymes and proteins and even lead to cell death [37]. Under normal conditions, ROS are constantly present in worms. However, factors such as medications, stress and illness can increase ROS levels [45,46]. ROS mainly targets DNA, proteins and lipids. Various drugs and natural products have been found to stimulate ROS production and trigger apoptosis, the process of programmed cell death [3,38]. Recently, the use of ROS-mediated apoptosis has emerged as an effective strategy to combat parasitic infections such as helminth parasites[38,47]. In this study, the use of *L*. *angustifolia* and *C*. *siliqua* EOs to treat fluke worms resulted in a dose-dependent increase in ROS levels in the worms. The NBT calorimetric assay showed a dose-dependent increase in ROS levels, suggesting that both *L*. *angustifolia* and *C*. *siliqua* EOs stimulated the production of ROS. This finding is consistent with previous studies using curcumin and thymoquinone to treat the liver fluke *Fasciola gigantica* [3].

The glutathione-dependent detoxification system, which involves GSH and GST, is considered a promising target for drugs and vaccines. These enzymes help bind reduced glutathione to pollutants, making them more water-soluble and easier to excrete from flukes [48,49]. Since both enzymatic and non-enzymatic molecules of the glutathione family are known to be essential for the antioxidant and detoxification processes of flukes, these molecules could be used to validate the effectiveness of new compounds or drugs[3]. GSH plays a crucial role in the cellular antioxidant defense mechanism, including maintaining the redox state by scavenging ROS. A decrease in GSH levels can lead to an imbalance in the redox process in parasites [50], as observed in our studies in flukes treated with both *L*. *angustifolia* and *C*. *siliqua* EOs, which has also been reported previously [3]. This disruption ultimately impacts intracellular redox homeostasis and impairs the worms’ ability to scavenge free radicals and electrophilic xenobiotics. The reduction in GSH activity after exposure to different concentrations of EOs from *L*. *angustifolia* and *C*. *siliqua* could be due to the destruction of antioxidant enzymes or the deficiency of minerals or vitamins [51]. As Baghbani et al. (2020) [51], showed excessive production of ROS and other free radicals can attack and damage protein molecules and antioxidant enzymes, reducing their activities. Studies also suggest that during oxidative stress, GSH-related enzymes, including GSH, consume glutathione to detoxify peroxides caused by the excessive production of ROS, leading to the depletion of its substrate [52]. In addition, GST has been reported to promote the development of parasite resistance to the widely used anthelmintics by catalyzing the conjugation of reduced glutathione via a sulfhydryl group to electrophilic sites of various substrates, constituting a phase II detoxification function [3]. Therefore, we wanted to investigate the possible effects of the EOs from *L*. *angustifolia* and *C*. *siliqua* on the GST molecule. A significant decrease in GST activity was observed in worms treated with EOs from *L*. *angustifolia* and *C*. *siliqua*, as also reported in the liver fluke *F*. *gigantica*[3]. Previous studies have shown that *L*. *angustifolia* and *C*. *siliqua* cause oxidative/nitrosative damage to biomolecules [20,21].

During *in vitro* treatment of *F*. *hepatica* with different concentrations of the EOs of *L*. *angustifolia* and *C*. *siliqua*, the parasites showed different responses. The use of EOs resulted in the production of ROS in the flukes, resulting in oxidative stress. In response, the flukes increased the activity of antioxidant enzymes, particularly SOD, to scavenge the ROS. This enzyme, together with other antioxidants, is responsible for catalyzing the dismutation of O_2_- to H_2_O_2_ [53], forming an effective system against ROS. However, when the flukes were treated with the highest concentrations of EOs from *L*. *angustifolia* and *C*. *siliqua* (25 and 50 mg/mL), this protective system was disrupted. Significant inhibition of SOD activity was observed in *F*. *hepatica*, possibly due to enzyme saturation caused by overproduction of hydroxyl ions and ROS. This renders the detoxification mechanism ineffective in *F*. *hepatica*, as has also been reported in other liver fluke species such as *G*. *explanatum* [54].

The quantitative and qualitative analyzes of oxidative DNA damage in living organisms can help to assess possible genotoxic influences. Reinecke and Reinecke (2004) [55] suggested using the comet assay as a biomarker for genotoxic influences on invertebrates. In our study, the results of the comet assay showed that the damage to *F*. *hepatica* DNA occurs in a concentration-dependent manner. Our results are consistent with a study by Rehman et al. (2020) [3], who found that exposure of adult *F*. *gigantica* to curcumin and thymoquinone also caused DNA damage.

This study has several limitations. Under *in vitro* conditions, the essential oils of *L*. *angustifolia* and *C*. *siliqua* were found to have anthelmintic properties against *F*. *hepatica*. However, it is important to note that no *in vivo* assessment of the *in vitro* findings was performed in this study. Despite their effectiveness, the use of these essential oils in the treatment of parasitic infections is currently limited due to a lack of comprehensive clinical trials and long-term safety data. There is currently no approved medication based on these essential oils that is used to treat parasites or other organic diseases. Therefore, further research is needed to fully understand their mechanisms of action, determine optimal dosing regimens, and evaluate potential side effects in human participants.

## Conclusion

In conclusion, the EOs of *L*. *angustifolia* and *C*. *siliqua* have shown promising fasciolicidal activity against fluke adults and eggs *in vitro*. Our study also suggests that these oils have an anthelmintic effect on *F*. *hepatica* by causing oxidative damage to biomolecules. The concentration of the oils plays an important role in their effects, as higher concentrations suppress *F*. *hepatica*’s antioxidant systems and damage lipids, proteins and DNA. Therefore, these two compounds could be further exploited to develop new drug formulations to combat helminth infections. However, further studies are required to better understand the functional significance of these compounds and their effects on parasites under *in vivo* conditions. This knowledge could ultimately lead to a sustainable approach to combating liver fluke infections.
